# Next-Generation Sequencing Insights into the Oral Microbiome and Antibiotic Resistance Genes in Grey Wolves (*Canis lupus*)

**DOI:** 10.3390/ani15243639

**Published:** 2025-12-17

**Authors:** Laura Sakarnyte, Renata Spinkyte, Lina Merkeviciene, Rita Siugzdiniene, Modestas Ruzauskas

**Affiliations:** 1 Microbiology and Virology Institute, Lithuanian University of Health Sciences, 44307 Kaunas, Lithuaniamodestas.ruzauskas@lsmu.lt (M.R.); 2 Department of Forest Sciences, Vytautas Magnus University, 44248 Kaunas, Lithuania; 3 Department of Anatomy and Physiology, Veterinary Academy, Lithuanian University of Health Sciences, 44307 Kaunas, Lithuania

**Keywords:** wolf, oral microbiome, *Canis lupus*, *Psychrobacter*, antimicrobial resistance

## Abstract

The oral microbiome of apex predators such as grey wolves (*Canis lupus*) is colonised by complex microbial communities and plays a crucial role in the health of wild mammals, but remains poorly understood. In this study, a single pooled sample mixed from oral samples of 17 wolves *(Canis lupus)* hunted in Lithuania was investigated for the determination of a variety of oral microbiota, providing the first metagenomic insight into wolf oral microbiomes in Baltic countries. The aim of this study was to identify the zoonotic and antimicrobial resistance potential of the oral microbiota. The results revealed diverse microbiomes associated with periodontal health as well as microbiomes related to the environment. Unique microbial taxa potentially reflect the nutritional and ecological interactions of carnivores. Shotgun metagenomic sequencing yielded a total of 18,726,406 raw reads, and following quality trimming and filtering, 86.01% of these (16,106,613) were retained. Among the total reads, 45.15% (8,455,255) were identified as host-derived and were removed. The most common oral bacterial genera were *Pseudomonas* (50%) and *Psychrobacter* (22.6%). Metagenomic reads for zoonotic pathogens, including *Salmonella*, *Mycobacterium* spp., *Yersinia*, *Coxiella burnetii*, *Corynebacterium pseudotuberculosis*, and others, were also detected, suggesting that grey wolves are potential natural reservoirs of zoonotic infections. Genes encoding antimicrobial resistance to many classes of antibiotics were also detected. This research contributes to understanding wolf dietary habits, oral health, the carriage and possible risk of transmitting AMR, and social interactions.

## Simple Summary

This study represents the first metagenomic analysis of the oral microbiome of grey wolves (*Canis lupus*) in Lithuania, revealing a diverse microbial community in a pooled sample from the oral cavity; the dominant bacterial genera were *Pseudomonas* spp. and *Psychrobacter* spp. Furthermore, metagenomic reads for several zoonotic pathogens were detected, including *Salmonella* spp., *Mycobacterium* spp., *Coxiella burnetii*, and others, suggesting that grey wolves may serve as potential carriers of zoonotic infections. Additionally, antimicrobial resistance (AMR) genes were identified, indicating potential environmental links to AMR spread across wildlife.

## 1. Introduction

The oral microbiome is a highly complex bacterial community that has a dynamic and diverse ecology and plays important roles in human and animal health [1]. Diverse and dynamic microenvironments are present in oral cavities and interact with the immune system of the host, thereby influencing both local and systemic immunity [2]. While the human oral microbiome has been extensively studied and compared with that of wild mammals, including in the context of antimicrobial resistance (AMR), the oral microbiome of grey wolves (*Canis lupus*) remains largely unexplored, and studies are limited. In humans, strong associations have been established between the oral microbiota and systemic diseases, highlighting the diagnostic and health relevance of microbiome profiling. Drawing parallels with human research, investigating the grey wolf oral microbiome can provide insights into host health, ecological exposure to AMR, and potential implications for conservation and zoonotic risk—areas that are not yet fully understood in wild animal populations [3,4]. As apex predators, wolves exert significant ecological influence by regulating prey populations and stabilising the ecological balance in the environment [5]. Grey wolves, as apex predators with wide-ranging behaviour, are bioindicators for environmental microbiome surveillance, including the spread of AMR and zoonotic pathogens [6]. Understanding the oral microbiomes of grey wolves can provide valuable information not only about their health status, behaviour, and physiology but also about potential microbial transmission at the wildlife–human interface [7]. This information may also elucidate potential antimicrobial resistance gene transfer between apex predators and environments and how oral microbial communities assemble, function, and adapt [8,9].

Wolves primarily consume a raw meat diet, with a preference for wild boar (*Sus scrofa*), roe deer (*Capreolus capreolus*), and, less frequently, moose (*Alces alces*) [10,11]. Dietary factors may contribute to the unique oral microbiota community, potentially harbouring pathogenic bacteria. Furthermore, wolves may be exposed to antibiotic-resistant bacteria through environmental contamination and dietary intake [5,10,11]. Antimicrobial resistance is a complex health problem that poses a significant threat not only to humans but also to wildlife and ecosystems [12]. According to the World Health Organization, bacterial resistance caused 1.27 million deaths globally in 2019 and was associated with nearly 5 million deaths overall [13]. The presence of antimicrobial resistance (AMR) genes in wildlife is often linked to anthropogenic pollution, with wild animals serving as potential reservoirs that contribute to the spread of AMR genes. Ongoing surveillance of AMR genes as well as zoonotic microorganisms in wildlife is essential for understanding transmission pathways and can assist in the development of effective One Health strategies [14,15,16].

Recent advances in next-generation sequencing have revolutionised microbiome research and provided unprecedented insights into microbial communities, enabling comprehensive analysis of microbial diversity [17]. Increasing evidence highlights the importance of profiling the entire microbiota rather than focusing solely on dominant bacterial taxa; these methods not only enable comprehensive microbiome profiling but are also widely applied for detecting AMR genes in the same datasets [18,19,20,21].

Analysis of the oral microbiome of grey wolves will enable not only the characterisation of the bacterial community composition and exploration of the prevalence of AMR genes within their unique oral microenvironment, but also the identification of specific AMR genes and zoonotic taxa of concern [22,23]. A diverse and balanced oral microbiome contributes to immune modulation and pathogen exclusion, which can influence both local oral health and broader systemic conditions [1,2,3,4]. Recent studies have demonstrated that the oral microbiota of grey wolves and wild bears (*Ursus arctos*) is influenced by factors such as altitude, climate, and human interaction. Key phyla, including Bacteroidetes, Proteobacteria, and Firmicutes, play significant roles in the systemic health, digestion, and immune system modulation of wild wolves [8,9]. Microbiomes vary significantly across animal populations, reflecting differences in the food chain, ecological niches, and human impact [7]. Despite some published data on the wolf microbiome, studies to date have focused on the gut microbiome. A diverse microbial ecosystem in different organ systems is unique, relatively stable, and resilient to environmental disturbance [24]. Moreover, a recent analysis of the free-ranging Swedish brown bear microbiome revealed that AMR in the oral microbiota aligns with historical patterns of antibiotic use. Since the composition and diversity of the microbiome play key roles in maintaining host health—through mechanisms such as immune modulation, pathogen exclusion, and metabolic support—alterations made by anthropogenic factors may have significant implications for systemic health in wildlife. Notably, the diversity of AMR genes has increased in parallel with antibiotic use in humans, reflecting the shared microbial landscape [8,9,21]. The data on antimicrobial resistance in wildlife can be influenced by anthropogenic activities, particularly farming and agriculture; therefore, this should be kept in mind during its interpretation.

The complex oral microbiota of healthy domestic dogs (*Canis lupus familiaris*), including *Porphyromonas* spp., *Fusobacterium* spp., *Neisseria* spp., and *Streptococcus* spp., has become a significant focus of microbiome research [25]. Studies have highlighted the potential of animal microbiomes as reservoirs of antibiotic resistance genes. Some bacterial taxa may contribute to periodontal disease, and other species can contribute to the natural oral microenvironment. This expanding field of study has revealed the ecological and evolutionary dynamics of the oral microbiota in domestic dogs and grey wolves and their broader implications for the spread of antimicrobial resistance across the environment and wildlife [26,27,28].

The aim of this study was to generate a metagenomic profile of the oral microbiome of wild grey wolves in Lithuania, with three main objectives: to characterise the taxonomical composition of the oral bacterial community; to assess the prevalence of diversity of AMR genes; and to identify, describe, and compare zoonotic taxa of potential concern in wildlife animals. The results are intended to establish a baseline for future wildlife microbiome surveillance with implications for animal health and zoonotic transmission within the One Health framework.

## 2. Materials and Methods

### 2.1. Animals and Sampling

**Figure 1:**
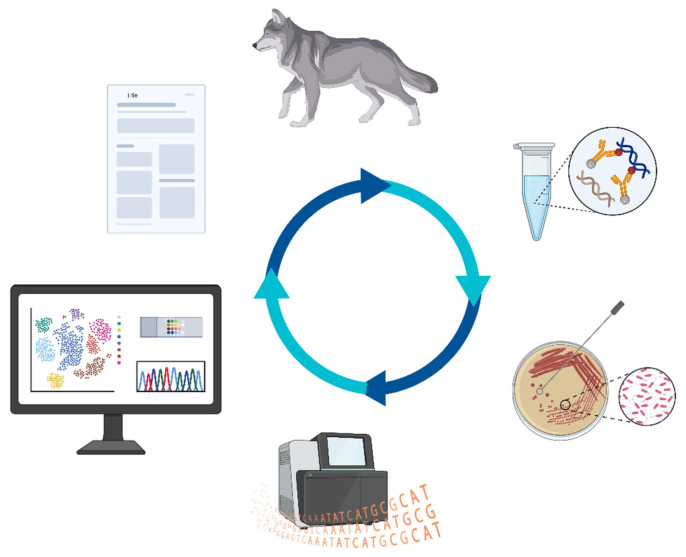
Circular workflow of microbiome sampling, sequencing, and analysis.

**Figure 2:**
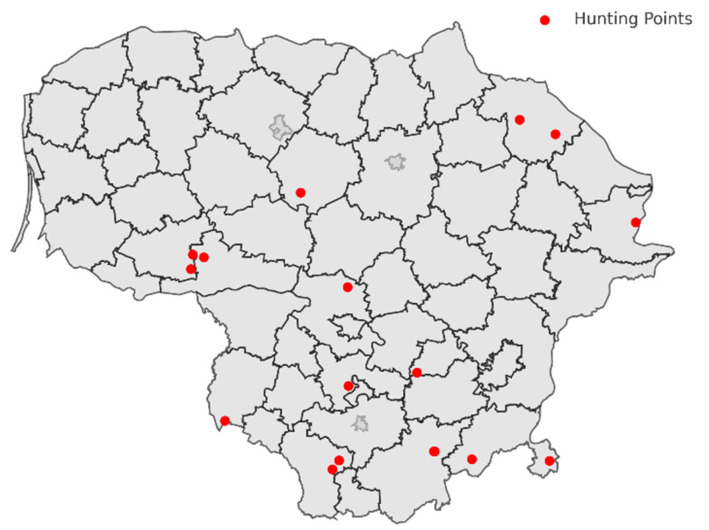
Geographic distribution of wolf hunting sites in Lithuania.

The grey wolves were hunted by a predetermined hunting quota set by the Ministry of Environment of Lithuania. All hunting activities were performed by hunters trained in the ethical harvest of animals for population regulation. No animals were hunted solely for the purpose of this research, and hunters’ decisions to harvest specific individuals were not influenced by participation in or knowledge of the research activities. All biological samples from freshly hunted animals were obtained in compliance with legally permitted hunting activities. The circular workflow of the research is provided in Figure 1. The samples were collected at 16 different locations throughout Lithuania (Figure 2), and detailed demographic and geographic data for the grey wolves are presented in Table 1 and Figure 2. All samples were collected from 2022 to 2024, specifically between 15 October and 1 April, during the hunting season.

Sampling was performed within a short period of time, which depended on the distance to the hunted places but took no longer than 2–6 h after hunting to ensure sample freshness and minimise environmental contamination. Before sampling, hunters were asked to maintain the hunted wolves in their initial positions with the aim of reducing the risk of contamination by ground. Samples were collected from the head side opposite the soil for more aseptic conditions. Sterile cotton swabs (Transwab; Medical Wire & Equipment Co., Ltd., Corsham, UK) were used tocollect clinical material from oral mucous membranes, manually exposing swabs close to the gingival margin while carefully avoiding contamination from other organs or external surfaces. Although cotton swabs are known to present certain limitations, they were chosen based on widespread clinical use, ease of handling in field conditions, and availability. Each swab was stirred and suspended in a DNA/RNA Shield Collection Tube (Zymo Research, Irvine, CA, USA) to preserve DNA integrity and delivered to the laboratory within 1–3 h. Even though samples were taken in quite a short period, the mucous membranes of some animals were dried; due to the low volume of material and keeping in mind the aim associated with data on zoonotic pathogens and resistance genes in the population of Lithuanian wolves, equal volumes of all samples (17 in total) were pooled into a single sample, placed into a 2 mL cryogenic storage tube, and stored at −80 °C until DNA extraction and metagenomic sequencing.

### 2.2. DNA Extraction and Next-Generation Sequencing

DNA extraction, quality control, library preparation, and next-generation sequencing (NGS) were performed, and the results were analysed via the Metagenomic Sequencing Service (Zymo Research, Irvine, CA, USA). DNA was extracted via a ZymoBIOMICS DNA Microprep Kit (Zymo Research) following the manufacturer’s protocol. Library preparation was conducted via a Nextera DNA Flex Library Prep Kit (Illumina, San Diego, CA, USA) with up to 500 ng of DNA input in accordance with the manufacturer’s instructions via internal dual-index 10 bp barcodes with Nextera adapters (Illumina). Thereafter, the libraries were quantified with TapeStation (Agilent Technologies, Santa Clara, CA, USA) and pooled in equal abundance. Due to the absence of dedicated funding for this project, samples were pooled prior to sequencing to reduce overall costs. While pooling samples limits individual-level resolution, it allowed for initial exploration of the oral microbiome and antimicrobial resistance profiles in wild grey wolves, which would not have been feasible otherwise. This approach provided a cost-effective strategy to generate data and provide background for more detailed investigations. The final pool was quantified via quantitative polymerase chain reaction, and the final library was subsequently sequenced on an Illumina NovaSeq system (Illumina, San Diego, CA, USA). The ZymoBIOMICS^®^ Microbial Community Standard (Zymo Research, Irvine, CA) was used as a positive control.

### 2.3. Bioinformatics and Data Analysis

The length of the sequence reads was 150 bp, and the sequencing depth was 17,790,085 reads. The quality of the DNA (A260/A280 ratio) was 1.619, and its concentration was 88.03 ng/µL. The raw sequence reads were trimmed to remove low-quality fractions and adapters with Trimmomatic-0.33 as described previously [28]. Quality trimming was performed via a sliding window with a 6 bp window size and a quality cut-off of 20, and reads with sizes smaller than 70 bp were removed. After that, host-derived reads were removed via Kraken2 [29] against the host genomes of *Canis lupus* and *Canis lupus familiaris*. Antimicrobial resistance gene identification was performed with the DIAMOND sequence aligner [30] against the NCBI AMR Database version 2020-08-09, with a mapping identity threshold >80% and a mapping coverage threshold >90%. If a read was mapped to multiple genes, it was assigned to the gene with the most occurrences. The microbial composition was profiled via sourmash [31] (k-mer size = 51). The GTDB species representative database (RS207) was used for bacterial and archaeal identification. The reads were mapped back to the genomes identified using sourmash via BWA-MEM [32], and the microbial abundance was determined using the counts of the mapped reads. The abundance cut-off was >10 mapped reads and a relative abundance >0.000001. The average library size was 467 bp, and Q30 was 95%. Host reads were removed using the Kraken2 tool. The taxonomic output was also visualised via Krona, generating an interactive plot that revealed the microbiome taxonomic distribution of the pooled sample. Visualisations were generated with BioRender.com, and Krona plots were generated via Krona for visualisation of the taxonomic composition.

## 3. Results

### 3.1. Sample Collection

Among the individuals sampled, 53% were female, and 47% were male, reflecting a relatively balanced sex ratio. Notably, first-year juvenile wolves comprised 53% of all samples, suggesting that younger individuals constitute a significant proportion. The remaining 47% of the wolves were older than 1 year.

### 3.2. Taxonomic Composition

Shotgun metagenomic sequencing via the Illumina HiSeq platform yielded a total of 18,726,406 raw reads. Following quality trimming and filtering, 86.01% of the raw reads (16,106,613) were retained. Among the total reads, 45.15% (8,455,255) were identified as host-derived and removed. Additionally, 1.71% (321,081) were classified as low-diversity sequences. The positive control revealed an appropriate quality of sequencing.

**Figure 3:**
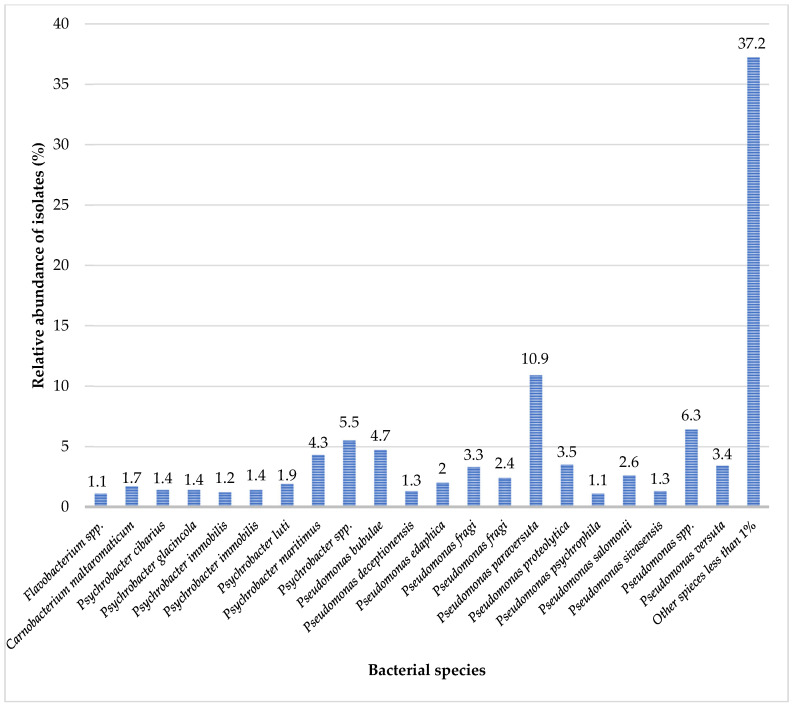
Taxonomic composition of the wolf oral microbiota at the species level. The bar chart displays the relative abundance of bacterial taxa, expressed as a percentage of total quality-filtered, non-host metagenomic reads assigned at the species level. Only species with a relative abundance > 1% are shown.

Taxonomic classification revealed that most reads (7,498,331) were assigned to bacteria (>98%), whereas smaller proportions represented Eukaryota (1%, 76,514 reads), Archaea (0.05%, 3,826 reads), viruses (0.04%, 3,061 reads), and unclassified reads (0.91%). The bacterial community was dominated by the phylum Proteobacteria (*Pseudomonadota*), accounting for approximately 50% of all bacterial reads. Bacteria of all classes (Alphaproteobacteria, Betaproteobacteria, and Gammaproteobacteria) were widely distributed among Pseudomonadota. The main orders among these Gram-negative bacteria included Enterobacterales, Hyphomicrobiales, and Nitrosomonadales. The bacterial species with relative abundances exceeding 1% are shown in Figure 3.

Metagenomic analysis of wolf samples revealed a taxonomically diverse oral microbiota. The most prevalent bacterial species identified were *Pseudomonas paraversuta* (10.9%), *Pseudomonas bubulae* (4.7%), *Psychrobacter maritimus* (4.3%), *Pseudomonas proteolytica* (3.5%), *Pseudomonas fragi* (3.3%), and *Pseudomonas versuta* (3.4%) (Figure 3). These six predominant species constituted approximately one-third of the bacterial community in the sample. The dominant phylum, comprising approximately 50% of the bacterial reads, was Proteobacteria from the family Pseudomonadaceae. In addition, the Miracellaceae family, primarily the genus *Psychrobacter*, was also prevalent in the oral microbiota. Other detected but less dominant taxa included *Flavobacterium* sp., *Carnobacterium maltaromaticum*, and others.

**Figure 4:**
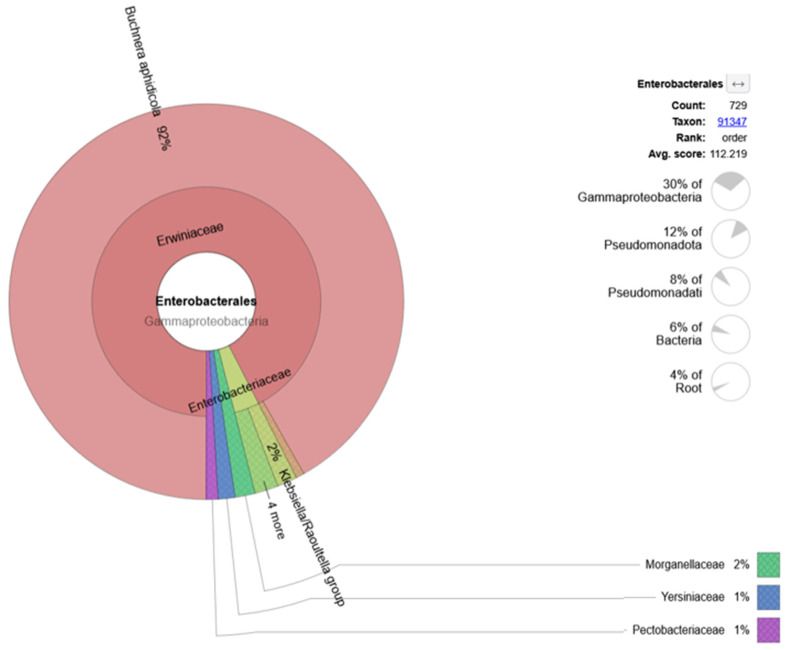
Relative abundance of bacterial families within Enterobacterales, based on taxonomic classification of quality-filtered, non-host metagenomic reads.

Bacteria of the order Enterobacterales comprised 30% of all Gammaproteobacteria (Figure 4). The main family within Gammaproteobacteria was *Erwiniacaea*, followed by *Enterobacteriaceae*, *Morganellaceae*, *Yersiniaceae*, and *Pectobacteriaceae*.

**Figure 5:**
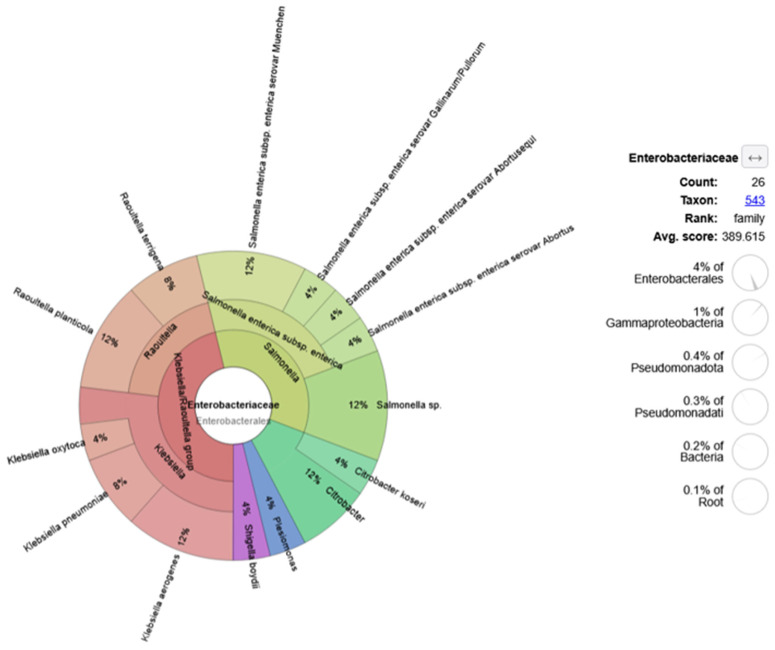
Relative abundance of Enterobacteriaceae, based on taxonomic classification of quality-filtered, non-host metagenomic reads.

A more detailed distribution of the genus Enterobacteriaceae is presented in Figure 5. Different *Salmonella enterica* serovars with a total of 26 reads (Muenchen, Gallinarum-Pulorum, Abortus, Abortusequi, and some non-subtyped) were found within this family. Other notable Enterobacteriaceae of zoonotic importance included *Klebsiella pneumoniae. K. oxytoca*, *K. aerogenes* (12 reads), *Shigella boydii*, and *Citrobacter* spp.

**Figure 6:**
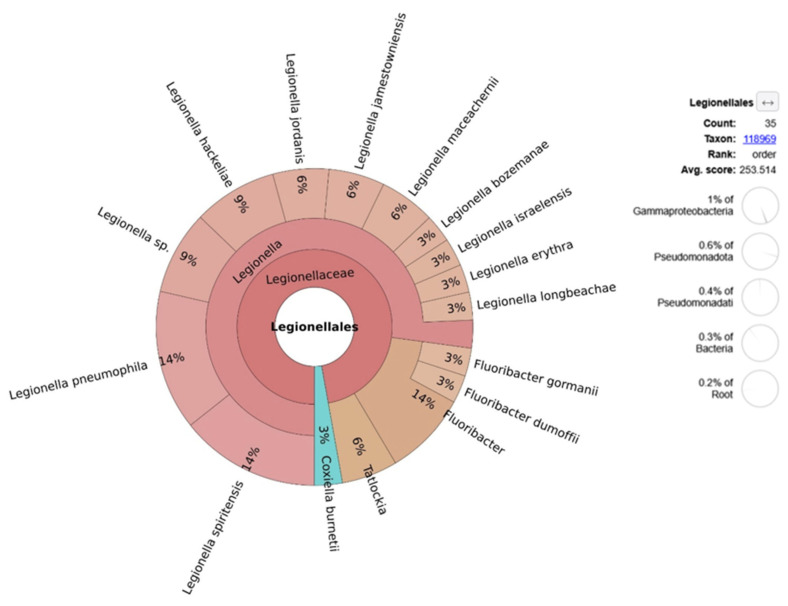
Relative abundance of bacterial species within Legionellales, based on taxonomic classification of quality-filtered, non-host metagenomic reads.

Bacterial taxa from the order Legionellales accounted for approximately 1% of the Gammaproteobacteria, with a total of 35 reads. Within this group, several *Legionella* species were identified, including *L. pneumophila* and *L. spiritensis*. Metagenomic reads consistent with *Coxiella burnetii* were also detected (Figure 6).

Furthermore, members of the order *Mycobacteriales* spp. were detected at low abundance, with a total of 17 sequence reads. This group included pathogenic species, such as *Mycobacterium leprea* and *Corynebacterium pseudotuberculosis*; these trace signals should not be excluded due to the low number of reads.

### 3.3. Metagenomic and AMR Genes Analysis

Metagenomic analysis revealed a broad spectrum of antimicrobial resistance genes across multiple antibiotic classes. As shown in Table 2, the genes encoding resistance to beta-lactams included extended-spectrum beta-lactamases, metallo-lactamases, carbapenemases, and carbenicillin-hydrolysing beta-lactamases. A wide spectrum of genes encoding resistance to tetracyclines was also detected. These genes included ribosomal protection proteins (*tetM*, *tetL*, *TetH*, and *Tet32*) and tetracycline efflux transporters, including MFS and ABC (*tetG*, *tetY*, and *tetE*). Notably, *Tet(34)* was identified, encoding phosphoribosyltransferase domain-containing proteins that cause resistance to oxytetracycline specifically. Resistance to aminoglycosides was expressed as the presence of aminoglycoside O-phosphotransferases (APHs) and nucleotidyltransferases (AadA). Additionally, resistance to rifampin was associated with both ADP-ribosyltranferases and rifampin monooxygenases (*Rox*, *Iri*). Resistance to glycopeptides included vancomycin resistance kinases (VanS) and the DNA-binding response regulator VanR. Resistance genes to other classes were also detected, including quinolones, macrolides, rifamycins, amphenicols, and sulphonamides. β-lactam resistance genes were the most diverse, accounting for nearly one-third of all detected genes. Detailed information about ARG numbers and potential taxa of their carriage is presented in Supplementary Table S1.

## 4. Discussion

As apex predators, grey wolves are ecologically important for maintaining balance and are sensitive to natural environmental disturbances [8]. In this study, we focused on oral bacterial composition, AMR genes, and zoonotic potential; the dominant bacterial phylum identified in the wolf oral microbiome was Proteobacteria (*Pseudomonadota*), accounting for approximately 50% of all bacterial reads. While comprehensive studies on the oral microbiota of wolves are limited, broader attention has been given to the gut microbiota of wild carnivores. For example, Wu et al. [9] analysed the gut microbiota of wolves and 16 other rare wild carnivores and reported that the bacterial phyla present were Bacteroidetes, Firmicutes, Fusobacteria, and Proteobacteria. These phyla are shared across wild wolves, domesticated wolves, and dogs. Additional studies have confirmed that the core gut microbiota in wild wolves typically consists of the bacteria mentioned above [8]. Furthermore, Lafferty et al. [33] revealed that in the wild American marten (*Martes americana*), the gut microbiota represents 45.31% Proteobacteria; this is comparable to the abundance observed in our wolf oral microbiome research, meaning that this phylum is highly abundant in the digestive tract, including the oral cavity, of carnivores. Notably, in our study, the abundance of the genus *Psychrobacter* was high; this taxon remains unexplored in wild carnivores, especially grey wolves, and further targeted studies are necessary to clarify its function in the oral niche. Previous reports have revealed the presence of *Psychrobacter pulmonis* in the lungs of lambs in Spain [34], as well as in polar bear (*Ursus maritimus*) faecal samples [35]. Notably, a recent study by Podar et al. [8] examined the oral microbiome across dogs, wolves, and humans. This research revealed that the oral microbial diversity of dogs is broader than that of wolves and humans. Despite their differences, dogs and wolves still share several taxa in their oral microbiota, which reflects their evolutionary relationships [9]. According to our previous study performed using the same diagnostic and data analysis methods, the most prevalent oral microbiota in healthy dogs in Lithuania were *Porphyromonas* spp., *Corynebacterium* spp., *Lampropedia* spp., and *Tannerella* spp. [36]. The detected differences in the oral microbiome of dogs and wolves may be explained by their different feed, living environment, and sanitary conditions (dry food at home versus hunted and dead animals; flesh contamination by soil particles). It should also be noted that the high abundance of bacteria prevalent in cold environments, such as *Psychrobacter* and *Pseudomonas*, in wolves can also be associated with post-mortem examination, as the samples were taken 2–6 h after the animals’ hunting.

Zoonotic infectious diseases pose a significant threat to human and public health [35]. Although grey wolves are typically absent from urban environments, these apex predators have direct and indirect contact with humans through overlapping ecosystems [37]. Recent studies have shown that zoonotic bacteria are detected in urban wildlife faeces [37]. Q fever from the causative organism *Coxiella burnetii* was detected in our study. Q fever is an emerging zoonosis with high environmental persistence. González-Barrio et al. [38] reported *Coxiella burnetii* in 109 wild mammal species; to our knowledge, our research reports it for the first time in grey wolves. The identification of *Coxiella burnetii* in this apex predator is relevant given its primary prey species, which are wild ungulates and wild boar [39,40]. The presence of *Coxiella burnetii* in this study should be viewed with caution, considering possible trace signals of a small number of reads. *Salmonella*, a true pathogen of multiple species of vertebrates, including humans, has also been detected in the oral cavities of wolves. Different serotypes probably have different origins and may include both small mammals (rodents) and large herbivorous species [22]. Markeviciene et al. [22] reported that in Lithuanian wild and domesticated reptile species, 68% of *Salmonella* isolates were resistant to at least one antimicrobial. The most frequent resistance of the isolates was against streptomycin (26%) [14]. Although *Salmonella* control programs in animal husbandry have been successful in Lithuania, this pathogen still circulates in wildlife—including predators. Such circulation ensures the survival of pathogens in the environment outside urbanised areas. It should also be noted that the total reads of *Salmonella*, *Klebsiella*, and other bacteria of the family Enterobacteriaceae were low; the prevalence of such bacteria may be due to a few individual animals, and therefore, more investigations should be carried out in the future to prove the carriage of certain pathogens in grey wolves.

Antimicrobial-resistant bacteria and their associated genes in wild apex predators remain largely understudied; however, a few previous studies have been conducted. Lee et al. [14] reported that compared with farmed cattle, wild mammals such as feral swine and coyotes often carry a greater burden of AMR bacteria and genes. These findings suggest that wild carnivores living in shared spaces exchange microbiota, including AMR bacteria and genes, which can interfere with livestock and potentially serve as reservoirs and vectors of AMR [22]. Wild carnivores, especially predators, play important roles in the One Health framework because of their potential to harbour AMR bacteria and genes. Di Francesco et al. [23] performed a molecular survey of 11 Apennine wolves in Italy and revealed the presence of AMR genes in various organs. This suggests that wolves may act as unnoticed reservoirs and spread resistance elements across ecosystems, given the extensive movement and apex predator status of wolves [23]. Apex predators and species such as gulls that migrate or scavenge in human-modified environments may facilitate the spread of resistance across regions [15]. Gonçalves et al. [6] analysed faecal samples from wolves (*Canis lupus*) and revealed high rates of resistance genes harboured by *E. coli* and *Enterococcus* spp. isolates (*tet*(M), *tet*(L), and *erm*(B) *bla*_TEM_; *tet*(A) and/or *tet*(B); and *aadA* or *strA*-*strB* genes). Despite their importance, wildlife is rarely included in AMR monitoring programs, leaving critical gaps in our understanding of AMR dynamics outside of veterinary, human medicine, and agricultural settings. Moreover, for more fundamental data, wildlife AMR monitoring needs longitudinal, non-pooled designs and environmental metadata. Integrating wildlife into surveillance systems offers a powerful opportunity to track the spread of environmental AMR [41].

Akwongo et al. [42] reported antimicrobial resistance in wild game animals. The authors revealed that antimicrobial-resistant bacteria such as *E. coli*, *Salmonella* spp., *Staphylococcus* spp., *Campylobacter* spp., *Listeria* spp., and *Yersinia* spp. are widespread among mammals worldwide. Nearly 60% of the isolates were resistant to at least one antibiotic, and approximately 17% of the isolates were resistant to more than one antibiotic. Resistance to antimicrobials was also detected in wildlife from remote, low-anthropogenic environments [42]. Wolves (*Canis lupus*) are often detected in remote, low-human-impact environments where wild game animals also reside [43]. Even in minimally disturbed ecosystems such as Madagascan forests, antibiotic resistance genes cross species and environmental boundaries [44]. This explains why apex predators, even those in natural habitats, are not isolated from the spread and circulation of AMR in the ecosystem.

In terms of prey composition, human interactions may influence the oral microbiome of wolves. Two observational studies conducted in Yellowstone National Park revealed that the oral microbiome of wolves is shaped by both social structure and host genetics. Wolves belonging to the same pack tend to have similar microbial communities, suggesting that social interactions and genetic relatedness contribute to the transfer of bacteria [9]. Furthermore, one study comparing wolves, domesticated dogs, and humans revealed that dogs, which consume omnivorous diets, exhibit greater oral microbial diversity compared with wolves, indicating that dietary differences can affect microbial diversity [45].

The dietary patterns of wolves across diverse European landscapes indicate a strong and consistent preference for wild ungulates. A study in Romania examining wolf feeding habits revealed that wolves rely mostly on wild boar (*Sus scrofa*), roe deer (*Capreolus capreolus*), and red deer (*Cervus elaphus*). Seasonal analysis revealed little change, with wild prey dominating year-round [46]. A. Otero et al. [47]’s research in Spain revealed that wolves consume feral horses (*Equus caballus*) and a low proportion of livestock and wild boars. Newsome et al. [43] suggested that the diet of wolves is dominated by large (240–650 kg) and medium-sized (23–130 kg) wild ungulates. A long-term study in Germany analysed wolf diets for more than eight years, confirming that wild ungulates constitute more than 96% of the diet of wolves, and this research highlights wolves’ minimal reliance on livestock [48]. These different feeding habits may explain the different oral compositions of the microbiota in different populations of wild animals within the same species.

Domesticated dogs (*Canis lupus familiaris*) exhibit notable dietary and physiological differences from grey wolves (*Canis lupus*). While wolves rely primarily on wild ungulates, dogs have adapted to a more omnivorous, starch-rich diet due to domestication and evolution. Wolves typically consume a high-protein, low-starch diet of wild ungulates, whereas domestic dogs subsist on starch-rich omnivorous diets [49,50,51]. For example, gut microbiome comparisons found higher microbial diversity in dogs than in captive wolves, illustrating diet-related microbial adaptation [50]. This pattern is mirrored in the oral cavity [8]. The oral microbiota of dogs consists mostly of *Porphyromonas a. cangingivalis*, *Porphyromonas gulae*, *Conchiformibius steedae*, *Porphyromonas gingivicanis*, *and Neisseria weaver*, which may be associated with their broader dietary habits and processed foods [26,49].

## 5. Conclusions

This study presents the first metagenomic characterisation of the oral microbiota of grey wolves (*Canis lupus*) in the Baltic region, with a specific focus on populations in Lithuania. The results revealed a microenvironment within the oral cavity consisting of a wide range of bacterial taxa associated with host health, environmental exposure, and dietary habits shaped by multiple ecological variables and possibly anthropogenic influences. The dominant bacterial phylum was Proteobacteria (*Pseudomonadota*), accounting for nearly 50% of all classified reads, followed by Myxococcota, Bacillota, and Spirochaetota at lower abundances. Notably, metagenomic reads consistent with zoonotic microorganisms such as *Coxiella burnetii* were also detected. To our knowledge, this represents the first report of the causative organism of Q fever in grey wolves. These findings suggest that environmental factors—such as human-induced ecological changes, dietary variation, and pollutant exposure—may contribute to shaping the oral microbiome of wild apex predators. The detection of metagenomic reads of zoonotic pathogens and AMR genes highlights not only potential health risks for wildlife but also their importance in One Health surveillance. Understanding the microbiota of wolves (*Canis lupus*) has important implications for understanding the role of wildlife in the ecology of antimicrobial resistance. In addition, antimicrobial resistance genes were identified, with the majority being resistant to β-lactams and tetracyclines. The determinants of AMR in wildlife within Baltic countries remain underexplored, especially in apex predators such as wolves, and these findings provide a baseline for future investigations involving more individualised analyses of grey wolves.

## Figures and Tables

**Table 1:** Overview of wolf samples collected across the Lithuanian districts.

Number	Date	Location, District	Sex	Juvenile/Adult
1	24 February 2023	Rokišis	♀	adult
2	24 February 2023	Rokišis	♀	juvenile
3	25 November 2023	Jurbarkas	♂	juvenile
4	2 December 2023	Radviliškis	♀	juvenile
5	4 December 2023	Varėna	♂	adult
6	4 December 2023	Varėna	♀	juvenile
7	4 December 2023	Jurbarkas	♀	adult
8	4 December 2023	Jurbarkas	♂	adult
9	11 January 2024	Lazdijai	♂	adult
10	13 January 2024	Kaunas	♂	juvenile
11	10 January 2024	Vilkaviškis	♂	adult
12	10 January 2024	Šalčininkai	♀	adult
13	21 January 2024	Šalčininkai	♂	adult
14	3 January 2024	Lazdijai	♂	juvenile
15	23 January 2024	Prienai	♀	juvenile
16	30 December 2024	Elektrenai	♀	juvenile
17	7 January 2024	Ignalina	♀	juvenile

**Table 2:** Genes encoding antimicrobial resistance detected in the oral microbiome of wolves.

Antimicrobial Class	Genes Encoding Resistance
β-lactams	class A beta-lactamase *BlaA* class A extended-spectrum beta-lactamase *CTX-M-165* class A extended-spectrum beta-lactamases *OXY-1*, *OXY5*, *OXY-6* RTG family carbenicillin-hydrolysing class A beta-lactamases *CARB-5*, *CARB-8*, *CARB-14*, *CARB-16*, *CARB-49* cephalosporin-hydrolysing class A beta-lactamase *ROB-1* class A beta-lactamase *FONA-3* class C beta-lactamase *CMY-72* class C beta-lactamases *EC-8*, *EC-13*, *EC-15*, *EC-16*, *ACT-42*, *ACT-44* class D beta-lactamases *OXA-209*, *OXA-275 OXA-335 OXA-551* putative metallo-beta-lactamase *SPR-1* *OXA-48* family carbapenem-hydrolysing class D beta-lactamase *OXA-416* *OXA-548* family class D beta-lactamase OXA-552
Tetracyclines	tetracycline resistance ribosomal protection proteins *Tet(M)*, *Tet(L)*, *Tet(H)*, *Tet(32)* tetracycline efflux Na+/H+ antiporter family transporter *Tet(35)* tetracycline efflux ABC transporter *Tet(46)* subunit B tetracycline efflux MFS transporters *Tet (G)Tet(Y) Tet(E)*, *Tet(33)*, *Tet(39)*, *Tet(41)* oxytetracycline resistance phosphoribosyltransferase domain-containing protein *Tet(34)*
Macrolides, lincosamides, and streptogramins	*Mph(E)* family macrolide 2′-phosphotransferase streptogramin An O-acetyltransferase *Vat(F)*
Aminoglycosides	aminoglycoside O-phosphotransferases *APH(3*″*)-Ia*, *APH(3″)-Ib* aminoglycoside O-phosphotransferase *APH(6)-Id* *ANT(3″)-Ia* family aminoglycoside nucleotidyltransferases *AadA5*, *A14*
Rifampin	NAD(+)–rifampin ADP-ribosyltransferase rifampin monooxygenases *Rox*, *Iri*
Chinolones	quinolone resistance pentapeptide repeat proteins *QnrB43*, *QnrD2* quinolone resistance pentapeptide repeat proteins *QnrB7*, *B33*, *B49*
Glycopeptides	*VanG*-type vancomycin resistance histidine kinase *VanS* *VanD*-type vancomycin resistance histidine kinase *VanS* *VanO*-type vancomycin resistance DNA-binding response regulator *VanR*
Amphenicols	type B-3 chloramphenicol O-acetyltransferases *CatB3*, *CatB8* chloramphenicol/florfenicol efflux MFS transporter *FloR*
Multidrug resistance	multidrug efflux RND transporter permeases *subunit OqxB3*, *4*, *7*, *8*, *12*, *22*, *23*, *26*, *27*, *28*, *30*, *31*
Other classes	streptothricin N-acetyltransferase *Sat2* sulphonamide-resistant dihydropteroate synthase *Sul2*

## Data Availability

The data are available upon request from the corresponding author. Metagenomic sequencing data are deposited in the NCBI repository (project number PRJNA1358942).

## Supplementary Materials

The following supporting information can be downloaded at: https://www.mdpi.com/article/10.3390/ani15243639/s1, Table S1: ARG in pooled oral sample of wolves.
